# Cardiologic Long-Term Follow-Up of Patients Treated With Chest Radiotherapy: When and How?

**DOI:** 10.3389/fcvm.2021.671001

**Published:** 2021-10-25

**Authors:** Chiara Lestuzzi, Maurizio Mascarin, Elisa Coassin, Maria Laura Canale, Fabio Turazza

**Affiliations:** ^1^Azienda Sanitaria Friuli Occidentale (ASFO) Department of Cardiology, Cardiology and Cardio-Oncology Rehabilitation Service, Centro di Riferimento Oncologico (CRO), Istituto di Ricerca e Cura di Carattere Scientifico (IRCCS), Aviano, Italy; ^2^Adolescents and Young Adults (AYA) Oncology and Pediatric Radiotherapy Unit, Centro di Riferimento Oncologico (CRO), Istituto di Ricerca e Cura di Carattere Scientifico (IRCCS), Aviano, Italy; ^3^Cardiology Department, Azienda Usl Toscana Nord-Ovest, Ospedale Versilia, Camaiore, Italy; ^4^Cardiology Unit, Istituto Nazionale Tumori (INT), Istituto di Ricerca e Cura di Carattere Scientifico (IRCCS), Milan, Italy

**Keywords:** radiotherapy—adverse effects, long term survivors, lymphoma, radiation-induced heart disease (RIHD), coronary artery disease, valvular heart disease (VHD), left ventricular dysfunction (LVD), cardiotoxicity

## Abstract

**Introduction:** Radiotherapy may cause valvular (VHD), pericardial, coronary artery disease (CAD), left ventricular dysfunction (LVD), arrhythmias. The risk of radiation induced heart disease (RIHD) increases over time. The current guidelines suggest a screening for RIHD every 5 years in the long-term survivors who had been treated by chest RT.

**Methods:** We reviewed the clinical and instrumental data of 106 patients diagnosed with RIHD. In one group (Group A: 69 patients) RIHD was diagnosed in an asymptomatic phase through a screening with ECG, echocardiogram and stress test. A second group (37 patients) was seen when RIHD was symptomatic. We compared the characteristics of the two groups at the time of RT, of RIHD detection and at last follow-up.

**Results:** Overall, 64 patients (60%) had CAD (associated to other RIHD in 18); 39 (36.7%) had LVD (isolated in 20); 24 (22.6%) had VHD (isolated in 10 cases). The interval between the last negative test and the diagnosis of moderate or severe RIHD was <5 years in 26 patients, and <4 years in 18. In group A, 63% of the patients with CAD had silent ischemia. The two groups did not differ with regard to type of tumor, cardiovascular risk factors, use of anthracycline-based chemotherapy, age at RT treatment, radiation dose and interval between RT and toxicity detection. The mean time from RT and RIHD was 16 years in group A and 15 in group B. Interventional therapy at RIHD diagnosis was more frequent in group B (54 vs. 30%, *p* < 0.05). At last follow-up, 27 patients had died (12 of cancer, 9 of cardiac causes, 6 of other causes); mean ejection fraction was 60% in group A and 50% in group B (*p* < 0.01). Patients with ejection fraction ≤ 50% were 14.5% in group A and 40% in group B (*p* < 0.01).

**Conclusions:** Clinically relevant RIHD become evident at a mean interval of 16 years after RT. The most frequent clinical manifestations are CAD and LVD. RIHD diagnosis in asymptomatic patients may preserve their cardiac function with timely interventions. We suggest -after 10 years from radiotherapy- a screening every 2–3 years.

## Introduction

Chest radiotherapy (RT) for mediastinal or lung tumors or also breast cancer (mostly left-sided), is associated with long-term cardiac adverse effects, namely coronary artery disease (CAD), valvular heart disease (VHD), left ventricular dysfunction (LVD), and pericardial disease (1, 2). The risk of radiation-induced heart disease (RIHD) increases over time: the cumulative incidence of RIHD requiring intervention, 20 years after mediastinal irradiation for Hodgkin's lymphoma, is 16% (3, 4). Survivors of childhood and adolescent cancer treated with RT, compared to their siblings, have a 5–6-fold risk of myocardial infarction, pericardial disease, or valvular abnormalities after 30 years of age (5). Cardiologic surveillance is, therefore, recommended every 5 years for cancer survivors treated with chest RTs, mostly for those treated during childhood, or when symptoms appear (6). We will analyze this approach on the basis of our experience at the CRO (National Cancer Institute of Aviano), in the cardio-oncology and long-term survivors clinic.

## Materials and Methods

We reviewed the clinical and instrumental data of 106 patients under care at the National Cancer Institute (CRO) of Aviano and who were diagnosed with RIHD. The study was approved by the internal review board. In one group (Group A: 69 patients), RIHD was diagnosed in an asymptomatic phase; these pertained to a group of 321 patients undergoing regular screening every 2–5 years with: clinical cardiologic examination, resting ECG, resting echocardiogram (M-mode, two-dimensional, and Doppler), and stress test for a period of 2–44 years (mean 17, median 16), or who were seen occasionally when referred to our outpatient cardiology clinic for routine examinations before surgery. A second group (Group B: 37 patients) was seen, due to complaints of symptoms related to their RIHD.

CAD was diagnosed in the presence of acute coronary syndrome or myocardial infarction or on the basis of provocative tests (treadmill or bicycle stress test, stress echocardiography, myocardial scintigraphy), and coronary angiography (7). LVD was diagnosed in the presence of a ≥15% drop in left ventricular ejection fraction (LVEF) from baseline to an absolute value of ≤ 53%, or a >10% drop to a value ≤ 50% (8, 9). Since a baseline echocardiogram before RT was not always available for patients treated before 1990, and in those treated in different hospitals, a LVEF of <45% was considered diagnostic of hypokinetic cardiomyopathy (10). The severity of valvular heart disease was assessed according to current guidelines at the time of the echocardiographic evaluation and integrated with cardiac catheterization and/or surgical data in patients who underwent cardiac surgery (11–13). We considered moderate to severe mitral regurgitation, aortic regurgitation, and/or aortic stenosis as being clinically relevant and considered VHD secondary to RT in the absence of other conditions (e.g., pre-existing valve abnormalities, severe mitral valve prolapse, clinical history of rheumatic heart disease, or bacterial endocarditis) that could be a possible cause.

## Statistical Analysis

Given the descriptive aim of the registry, no formal statistical design was set up. Descriptive data are presented as a percentage of the entire number of patients. Time to RIDH was calculated from radiotherapy to the first evidence of cardiac toxicity, while follow-up time was calculated from the time of RIDH to the last visit or death. Continuous variables were expressed as the mean ± standard deviation of and their differences were tested for significance with the Student's *t*-test. The association between clinical parameters were calculated using contingency table methods and tested for significance using the Pearson's chi-square test. All significance levels were set at a 0.05 value, and *p*-values were two-sided. SPSS software (version 19.00, SPSS, Chicago) was used for all statistical analysis.

## Results

### Demographics and Treatment Data

The patients were 34 males and 72 females, 8–67 years of age at the time of RT, with 78 who received mediastinal RTs for Hodgkin's lymphoma (*n* = 54) or Non-Hodgkin's lymphoma (*n* = 24), and 27 chest RTs for breast cancer (26 left-side, 1 right-side, including sternum in the RT plan). Cardiovascular risk factors included diabetes in 8 patients, dyslipidemia in 31, hypertension in 12, smoking habits in 4, and family history of CAD in 20. For all patients, an attempt was made to prevent all modifiable cardiovascular risk factors, such as encouraging them to perform regular physical activity, to avoid or stop smoking, and to regularly check blood glucose and lipids (14). To patients with hypertension, diabetes, and dyslipidemia detected during follow-up, appropriate medical therapy was also prescribed: anti-diabetics, statins and/or angiotensin inhibitors or beta-blockers (according to heart rate), and acetylsalicylic acid. In 6 patients, the total radiotherapy dose delivered was unknown. In the others, it ranged from 15 to 60 Gy; a >35 Gy dose—which doubles the risk of RIHD compared to doses of 20–30 Gy (15)—was administered to 77 patients. Anthracycline-based chemotherapy (before or after RT) was given to 68 patients (Table 1). Females were represented more in the asymptomatic group (75 vs. 54%, *p* <0.05). The two groups did not differ with regard to type of tumor, cardiovascular risk factors, use of anthracycline-based chemotherapy, age at RT, and radiation dose (Table 1). The RT techniques changed over time (with the extended mantle field RT progressively replaced in the 1990s by modern techniques, such as Involved Fields Radiotherapy IFRT, which significantly reduce radiation burden to the heart and the risk of RIHD) (16–21). We, therefore, also took this variable into consideration. The patients were treated between 1974 and 2010: 79 up to and 27 after 1999. The proportion of patients treated before 2000 (when modern techniques were introduced as standard treatment in our hospital), were similar in the two groups (Table 1). The only difference between the two was an increased prevalence of females in the asymptomatic group.

**Table 1:** Baseline characteristics of the entire study group and of the two groups.

	**Total ** **(*n =* 106)**	**Asymptomatic group ** **(*n =* 69) (%)**	**Symptomatic group ** **(*n =* 37) (%)**	***p***
Males	34	17 (25%)	17 (46%)	<0.05
Females	72	52 (75%)	20 (54%)	
Hodgkin's	54	33 (48%)	21 (57%)	NS
Non-Hodgkin's	24	15 (22%)	9 (24%)	NS
Breast cancer	27	20 (29%)	7 (19%)	NS
Diabetes	8	4 (5.8%)	4 (11%)	NS
Dyslipidemia	31	19 (27.5%)	12 (32%)	NS
Hypertension	12	7 (10%)	5 (13.5%)	NS
Active smoker	4	1 (1.5%)	3 (8%)	NS
Anthracyclines	79	52 (75%)	27 (73%)	NS
Total dose ≥35 Gy^*^	84/99^*^	56/63^*^ (88%)	28/36^*^ (78%)	NS
Treated before 2,000	79	51 (74%)	28 (76%)	NS
	**Mean** **±** **SD**	**Mean** **±** **SD**	**Mean** **±** **SD**	
Age at RT (years)	38 ± 14.7	39 ± 15	35 ± 15	NS
Radiation dose (Gy)	40 ± 8	41 ± 8	39 ± 9	NS

*RT, Radiotherapy; Gy, Grays; RIHD, Radiation Induced Heart Disease; SD, Standard Deviation.*

* *Data of 7 patients are missing; percentage calculated on the number of patients with complete information*.

### Radiation-Induced Heart Disease

**Figure 1:**
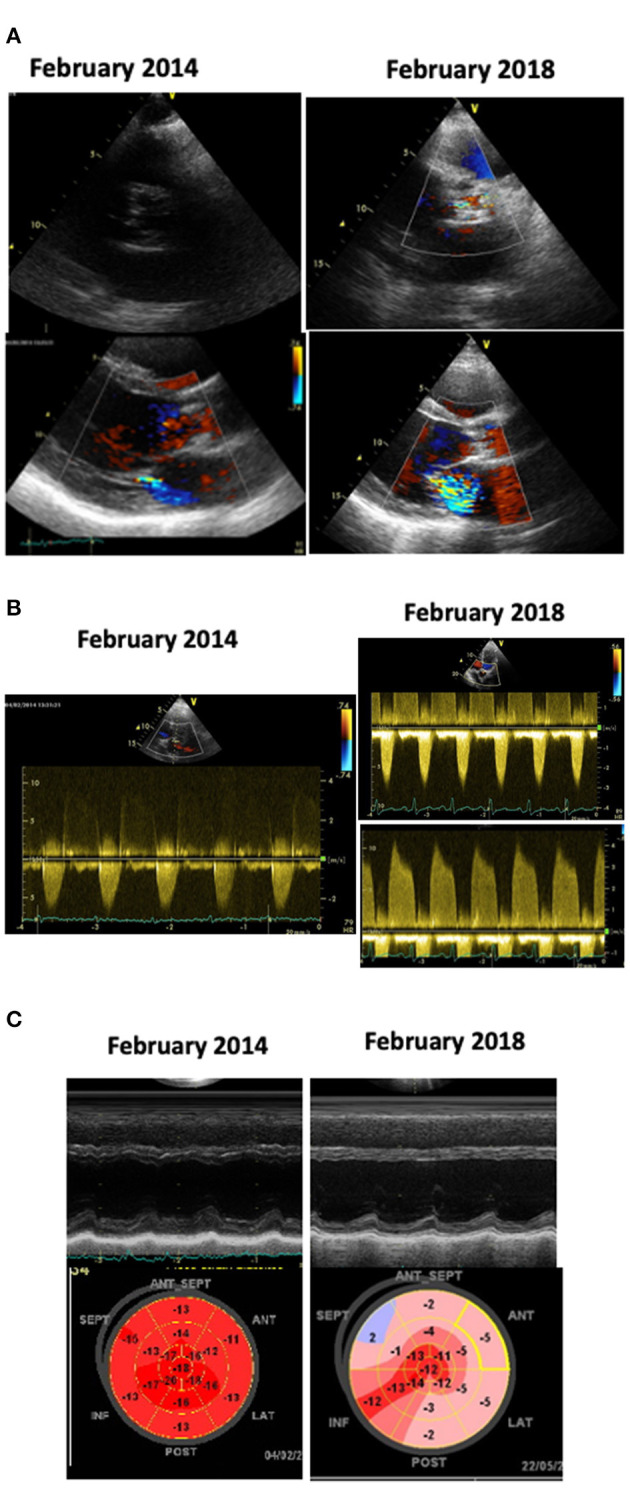
Echocardiograms of a patient treated with anthracyclines chemotherapy and mediastinal RT in 1999, at age 19. He underwent a cardiologic follow-up every 2–3 years. In February, 2014 (images on the left) the echocardiogram detected the new appearance of mild aortic and mitral dysfunction **(A,B)**; the left ventricular (LV) function and Global Longitudinal Strain (GLS) were normal **(C)**; a stress test was negative. We planned yearly check-up, but the patient, who felt completely asymptomatic, skipped the appointments. In February 2018, at age 38, he suddenly experienced a congestive heart failure. The echocardiogram (images on the right) showed: calcific aortic stenosis, severe mitral regurgitation **(A)**; severe aortic stenosis and moderate aortic regurgitation **(B)**; severe LV dysfunction and abnormal GLS **(C)**. At coronary angiography a 70% stenosis of the left anterior descending coronary artery was detected.

Overall, 64 patients (60%) had CAD (isolated in 46, associated to other cardiac diseases in 18); 39 patients (36.7%) had LVD (isolated in 20); 24 (22.6%) had valvular heart disease (isolated in 10 cases). Among patients in the asymptomatic group who had CAD diagnosed with a stress test, 21/32 (63%) had silent ischemia. Isolated LVD was more frequent in the asymptomatic group (23vs. 11%), but the difference was not statistically significant. On the contrary, the association between LVD and myocardial ischemia was significantly more frequent in the symptomatic group (22 vs. 3%, *p* <0.01). Isolated VHD and pericardial constriction were only detected in asymptomatic patients, while the association of cardiac ischemia and VHD, with or without LVD, was only observed in the symptomatic group. However, due to the small number of cases, the difference was not statistically significant (Table 2). In 17 patients with different manifestations of RIHD, a second or third toxicity was diagnosed, 1–18 (median of 7) years apart. The first diagnosis of RIHD was reached at a mean and median time of 16 years from RT (range 0–35 years), without any significant difference between the two groups. RIHD was diagnosed in 5 patients (1 in the asymptomatic group, 4 in the symptomatic group) in the first year after RT. All had been previously treated with anthracyclines, had a normal EF after chemotherapy, and developed LVD shortly after a mediastinal RT with ≥40 Gy. We, therefore, considered that, although these patients might have had subclinical anthracycline myocardial damage, the role of RT was relevant. The mean and median ages at the time of the first clinical evidence of RIHD were 54 and 52 years, respectively. Thirty-six patients presented one or more normal or minimally altered tests relevant to the specific RIHD (e.g., an echocardiogram for LVD and VHD, a provocative test for CAD), obtained before the diagnosis of moderate to severe disease (Figure 1). The interval between the last negative test and the diagnosis of moderate or severe RIHD was 0 (2 patients experienced an acute myocardial infarction a few months after a negative treadmill stress test) to 7 years (mean and median time of 3 years). In 26 patients (72%), the interval was <5 years; in 18 (50%) it was <4 years. Among patients with CAD, 22 had a previous negative stress test performed 0–6 years before (at a median time of 3 years); 3 who presented an acute myocardial infarction had a negative stress test within 2 years before the myocardial infarction.

**Table 2:** Characteristics of the patients at time of detection of radiation induced heart disease.

	**Total ** **(*n =* 106) (%)**	**Asymptomatic group ** **(*n =* 69)**	**Symptomatic group ** **(*n =* 37)**	***p***
Left ventricular dysfunction (LVD)	20 (19%)	16 (23%)	4 (11%)	NS
Cardiac ischemia	46 (43%)	33 (48%)	13 (35%)	NS
Valvular disease	10 (9%)	10 (14.5%)	0	NS
Pericardial constriction	1 (0.9%)	1 (1.5%)	0	NS
LVD + ischemia	10 (9%)	2(3%)	8 (22%)	0.01
Valvular disease + constriction	1 (0.9%)	0	1 (3%)	NS
LVD + valvular disease	5 (4.7%)	2 (3%)	3 (8%)	NS
Ischemic and valvular disease	4 (4%)	0	4 (11%)	NS
LVD + ischemia + valvular disease	4 (4%)	0	4 (11%)	NS
	**Min, Max, Median**	**Mean** **±** SD^*^	**Mean** **±** SD^*^	
Age at first RIHD detection (years)	22, 82, 52	55 ± 12	52 ± 13	NS^*^
Time from RT (years)	0, 35, 16	16 ± 9	15 ± 9	NS^*^
Interval between a normal test and RIHD	0, 23, 3	4 ± 3.7	3 ± 3.4	NS^*^

*LVD, Left Ventricular Dysfunction; PCI, Percutaneous Coronary Intervention; RT, Radiotherapy; SD, Standard deviation; NS, non-significant.*

* *P calculated on the mean ± SD*.

### Treatment and Follow-Up

After diagnosis, each patient was treated according to type and severity of their disease and symptoms. Cardiac surgery (either valvular or coronary artery graft), or interventions, such as Transcatheter Valve Replacement (TAVR) or Percutaneous Coronary Intervention (PCI), were indicated in 41 patients (38.7%). The need of interventional therapy was more frequently considered in the symptomatic group (54 vs. 30%, *p* < 0.05). After diagnosis, 11 patients (7 in the asymptomatic group and 4 in the symptomatic group) were lost to follow-up. For those in the asymptomatic group, a mean follow-up of 10 ± 5 years was available; for those in the symptomatic group, the available follow-up was 7 ± 4 years. Overall, 27 patients died (29% of the asymptomatic group and 20% of the symptomatic group—Table 3). The causes of death were cardiac-related (heart failure or acute myocardial infarction) in 9 cases, cancer progression or second/third cancer diagnosis in 12, sepsis in 2 cases, and unknown in 4. The left ventricular EF at the last follow-up was 60 ± 10% in the asymptomatic group and 50 ± 13% in the symptomatic group (*p* < 0.01). Patients with an EF <50% were 14.5% in the asymptomatic group and 40% in the symptomatic group (*p* < 0.01).

**Table 3:** Treatments and outcome after diagnosis.

		***Total* (*n* = 106) (%)**	**Asymptomatic group (*n* = 69)**	**Symptomatic group (*n* = 37)**	***p***
Cardiac surgery or PCI		41 (38.7%)	21 (30%)	20 (54%)	0.025
Mean follow-up after diagnosis (years)			10 ± 5	7 ±4	
Death		27	20 (29%)	7 (20%)	NS
Cause of death	Cardiac	9	1	8	
	Cancer	12	4	8	
	Others/unknown	6	3	3	
EF at last follow-up (mean value ± SD)			60 ± 10	50 ± 13	0.024
Patients with EF <50% at last follow-up		25 (23.6%)	10 (14.5%)	15 (40%)	0.01

*EF, Ejection Fraction; PCI, Percutaneous Coronary Intervention; SD, Standard Deviation*.

## Discussion

Our experience confirms previous reports assessing that the prevalence of moderate to severe valvular disease, CAD, and LVD in patients treated with mediastinal or chest RT is high and increases with time after irradiation (22–26). According to these observations, surveillance should be lifelong.

Our retrospective study included patients of different ages, with different tumors and under various radiation treatments. These differences might actually influence the incidence of RIHD in different subgroups (19). The number of patients was too low to allow for a comparison between mediastinal and breast RT, and the aim of the study was only to assess what the best approach for screening might be in patients at risk of RIHD in the real world, in a cardiology clinic, or in general clinical practice.

As previously reported, RT is an independent risk factor of CAD, and the disease is often asymptomatic: this warrants an active screening process (27, 28). Autonomic dysfunction, which is frequent after RT, is similar to the cardiac autonomic cardiopathy observed in diabetes, possibly secondary to direct cardiac nerve damage by RT, and might explain the absence of angina (29–31). In fact, most of our patients with CAD who were in the screening group had silent ischemia, and those in the symptomatic group had a higher prevalence of LVD (with dyspnea as a prevalent symptom). The problem of silent ischemia is particularly relevant because patients have no warning symptoms during physical exertion, and the first symptomatic episode may be an acute myocardial infarction, or an ischemic cardiomyopathy, which may lead to a chronic anatomic and functional defect (32). Therefore, regular screening for cardiac ischemia is highly recommended, regardless of the presence or absence of angina. Screening may be performed as it is for CAD in diabetic patients, using either a physical or pharmacological stress test (preferably with imaging, such as echo-stress, or with myocardial scintigraphy), computed tomography calcium score, or other methods (according to the availability of the tests in a given center), as well as balancing diagnostic utility, and cost and risk for the patient (33–36).

The time to progression of VHD and CAD was short in our patients, in contrast with a paper by Donnellan, who found a similar rate of progression in the aortic stenosis gradient in patients with or without previous RT (37). However, follow-up in the Donnellan study was shorter (lasting an average of 3.6 years) than in the present study, and the RT patients still had a more severe change in the aortic valve area and a significantly shorter time from the baseline echocardiogram to symptom onset and aortic valve replacement (AVR). The progression from aortic sclerosis to severe calcific stenosis involves genetic factors, lipoprotein deposition and oxidation, and chronic inflammation. The use of aggressive therapy to lower blood pressure, blood lipids, and to contrast chronic inflammation may slow down this process (38–43). According to our experience, patients who had any sort of aortic or coronary calcification detected during screening (even if subclinical), should undergo a strict follow-up (yearly or every other year), since the disease might worsen in a short period of time.

The higher prevalence of symptomatic patients undergoing valve replacement or revascularization in the present study is explained by the fact that, among the asymptomatic patients, major interventions were only proposed to those with severe disease or those at a high risk of clinical instability (e.g., critical stenosis of a main coronary artery or very severe aortic stenosis), while aggressive medical therapy with strict follow-up and additional tests (such as stress echocardiography or myocardial scintigraphy) were proposed to the others, in order to delay the need for cardiac surgery, TAVR, and/or PCI, which has often been reported to be technically difficult, risky, and with less probability of long-term success in these types of patients (44–51). Another reason to postpone surgery in the asymptomatic patients is that CAD and VHD may often progress at a different rate, requiring further interventions years apart, and we attempted to prevent re-surgery (52).

In terms of EF, the better outcome at follow-up of the asymptomatic patients could be explained by the fact that a timely therapeutic intervention (lowering blood pressure, prescribing statins, anti-inflammatory medication, and anti-ischemic therapy in these patients if needed, as well as performing cardiac surgery or percutaneous interventions for severe valvular disease or CAD) prevented myocardial infarctions and adverse cardiac remodeling, secondary to cardiac ischemia and myocardial fibrosis in patients with CAD, or to pressure overload in patients with VHD (53–56). Therefore, our experience reinforces the concept that RIHD should be recognized and treated before the symptomatic phase. A major problem, which is mostly detected in younger patients, is the fact that they are often reluctant to consider their cardiovascular risk and, therefore, might not adhere to prescriptions, as a reaction to post-traumatic stress, which may lead to denial (57–59).

With regard to the timing of screening tests, it is well-known (through large cohort studies) that the incidence of symptomatic RIHD is very low in the first 10 years after RT and increases rapidly afterwards. This is not limited to patients treated in adult age (who could have a risk linked to their age) but also to those treated in childhood who develop CAD or VHD at a relatively young age. Nevertheless, current guidelines suggest screening every 5 years or when symptoms develop, regardless of the time from RT. According to our experience, an interval of 5 years is too long, since many patients might progress from mild to severe disease during this period, possibly with the event of an acute myocardial infarction or sudden death. Moreover, symptoms such as dyspnea (secondary to VHD or angina equivalent) may be under-assessed and misinterpreted in patients with chronic lung dysfunction, as patients treated with chest RT frequently are (mostly if chemotherapy with bleomycin/or anthracycline were added) (60–63).

Along with regular screening tests, special attention must be given to these patients in relation to their adherence to suggested lifestyles and pharmacologic prescriptions. This behavior must be constantly reinforced. Since oncologists often dismiss patients from follow-up after a time span of 10–15 years from complete recovery, this should be carried out by other physicians: usually general practitioners who tend to their patient for all their various conditions, and cardiologists who conduct the follow-ups. Communication must be tailored to the particular psychological attitude of long-term cancer survivors (64).

## Conclusions

RIHD is an elusive clinical entity in the pre-symptomatic phase and can worsen dramatically in a short period of time. The timely recognition of subclinical RIHD and promptly prescribed therapies may improve the long-term outcome of patients who, after recovering from cancer, are at risk of cardiac events. Screening tests should be more frequent (every 2 or 3 years) after 10 years from RT, and even more frequent (on a yearly basis) in patients with a possible high risk of progression (initial valve disease, coronary calcification, moderate to high risk of CAD). General practitioners and general cardiologists (who may see patients for reasons that do not depend on their cancer history but just for routine check-ups), should be aware of the risk of RIHD, of its often elusive clinical presentation, of the need for and method of screening it, and should care for the many patients who are not followed by a long-term survivors clinic or by an oncocardiologist.

## Data Availability Statement

The raw data supporting the conclusions of this article will be made available by the authors, without undue reservation.

## Ethics Statement

The studies involving human participants were reviewed and approved by the Comitato Etico Unico Regionale (CEUR) Friuli Venezia Giulia. Written informed consent for participation was not required for this study in accordance with the national legislation and the institutional requirements.

## Author Contributions

CL, MM, and EC planned the study, recruited and followed the patients, and wrote the paper. MC performed the statistical analysis and contributed in writing the paper. FT contributed in writing the paper. All authors contributed to the article and approved the submitted version.

## Conflict of Interest

The authors declare that the research was conducted in the absence of any commercial or financial relationships that could be construed as a potential conflict of interest.

## Publisher's Note

All claims expressed in this article are solely those of the authors and do not necessarily represent those of their affiliated organizations, or those of the publisher, the editors and the reviewers. Any product that may be evaluated in this article, or claim that may be made by its manufacturer, is not guaranteed or endorsed by the publisher.
